# Timing of mTORI usage and outcomes in kidney transplant recipients

**DOI:** 10.7150/ijms.53655

**Published:** 2021-01-09

**Authors:** Lee-Moay Lim, Lan-Fang Kung, Mei-Chuan Kuo, A-Mei Huang, Hung-Tien Kuo

**Affiliations:** 1 Division of Nephrology, Department of Internal Medicine, Kaohsiung Medical University Hospital, Kaohsiung Medical University, Kaohsiung, Taiwan.; 2 Graduate Institute of Clinical Medicine, College of Medicine, Kaohsiung Medical University, Kaohsiung, Taiwan.; 3 Faculty of Medicine, College of Medicine, Kaohsiung Medical University, Kaohsiung, Taiwan.; 4 Department of Nursing, Kaohsiung Medical University Hospital, Kaohsiung, Taiwan.; 5 Faculty of Renal Care, College of Medicine, Kaohsiung Medical University, Kaohsiung, Taiwan.; 6 Graduate Institute of Medicine, College of Medicine, Kaohsiung Medical University, Kaohsiung, Taiwan.; 7 Department of Biochemistry, School of Medicine, College of Medicine, Kaohsiung Medical University, Kaohsiung, Taiwan.

**Keywords:** kidney transplantation, immunosuppressant, mammalian target of rapamycin inhibitor (mTORI), outcomes, malignancy

## Abstract

The introduction of mammalian target of rapamycin inhibitors (mTORi) as immunosuppressive agents has changed the landscape of calcineurin inhibitor-based immunosuppressive regimens. However, the timing of mTORi conversion and its associated outcomes in kidney transplantation have conflicting results. This study investigated the effect of early or late mTORi post-transplant initiation on major transplant outcomes, including post-transplant malignancy, in kidney transplant recipients in our center. We enrolled 201 kidney transplant recipients with surviving function grafts of >3 months between 1983 and 2016. Patients were divided into three groups: early mTORi (initiated within 6 months of kidney transplantation), late mTORi, (mTORi initiation >6 months after kidney transplantation) and no mTORi. The mean creatinine at conversion was 1.46 ± 0.48 mg/dL and 1.30 ± 0.53 mg/dL for the early and late mTORi groups, respectively. During the study period, 10.5% of mTORi users and 19.2% of mTORi nonusers developed malignancy, mainly urothelial carcinoma. After adjustment for confounding factors, mTORi users were found to have a lower incidence of post-transplant malignancy than did nonusers (adjusted OR: 0.28, *P* = 0.04). No significant difference was observed between early and late mTORi users. Our results verified the potential advantages of mTORi usage in reducing cancer incidence after kidney transplantation. However, no significant result was found related to the timing of mTORi introduction. Future studies should include a longer observation period with a larger cohort.

## Introduction

Advancements in surgical and medical techniques and immunosuppressive agents have led to remarkable short-term outcomes in kidney transplantation. However, with improved patient survival and an aging transplant population, long-term complications have become a major challenge 1. The prolonged modification of the immune system of kidney transplant recipients is associated with the risk of opportunistic infection and various cancers 1.

The incidence of malignancy in kidney transplant recipients is at least 2-4-fold higher than that in the age- and sex-matched general population 1, 2, with higher cancer-related mortality 3. The pattern of post-transplant malignancy (PTM) varies among geographical regions. Studies from Western countries have indicated that non-melanoma skin cancer is the most common PTM 4. By contrast, skin cancer has a lower incidence in the Asian population. In Japan, gastric and kidney cancer are the two most frequent PTMs, whereas in Korea, they are gastric cancer and lymphoma 5, 6. In Hong Kong, non-Hodgkin lymphoma has the highest standardized incidence ratio among PTMs 7, and in Taiwan and China, urothelial carcinoma is the most common PTM (approximately 40%) 8, 9.

Immunosuppressive therapies may directly affect cancer growth through various mechanisms. Cyclosporin and tacrolimus increase the risk of malignancy in kidney transplant recipients 10, 11, whereas mammalian target of rapamycin inhibitors (mTORi) reduce the risk 12, 13. However, mTORi use in kidney transplantation is associated with inferior graft survival 14, 15 and increased mortality risk 16, 17. In recent meta-analysis, Wolf S. et al found that initiation of mTORi within 3 months of kidney transplantation may reduce the future risk of malignancy 18 but in Hahn et al. analysis, mTORi treatment failed to demonstrate a reduction in cancer risk 19. These controversial results regarding the role of mTORi and timing of introduction in transplant outcomes need further evidence for verification.

This study investigated the effect of early and late mTORi initiation on major transplant outcomes, including PTMs, in kidney transplant recipients.

## Materials and Methods

### Study population

This retrospective observational study included kidney transplant recipients followed up in a medical center in southern Taiwan from January 1983 to April 2016. The immunosuppressive treatment in our hospital consisted of induction therapy (methylprednisolone and basiliximab, an interleukin-2 receptor antagonist) and maintenance treatment (based on tacrolimus, mycophenolate mofetil, and prednisolone). mTORi, including rapamycin and everolimus, were included in the maintenance regimen if the graft function was stable 3 months after transplantation, minimizing the calcineurin inhibitor (CNI) dosage. Inclusion criteria were kidney transplant recipients who had mTORi as one of their maintenance immunosuppressive agents with a treatment duration of >3 months. We excluded patients who were diagnosed with malignancy before the introduction of mTORi. A total of 201 kidney transplant recipients who survived with functioning grafts for >3 months were included.

At the baseline visit, the patients' sociodemographic characteristics, medical history, and current maintenance immunosuppressive medications were recorded. Their medical histories were confirmed through doctors' chart reviews. The patients' biochemistry measurements were collected at the baseline visit, before and after kidney transplantation, and at the beginning of mTORi treatment. The patients were stratified into three groups according to mTORi usage and time of initiation: no mTORi, early mTORi (mTORi initiation within 6 months of kidney transplantation), and late mTORi (mTORi initiation >6 months after kidney transplantation). Our patients received regular follow-up in the nephrology and urology clinics after the transplant surgery for immune status and disease progression monitoring. Screening for malignancy, including tumor marker survey, kidney and liver sonography, chest X-ray, and urine cytology, was conducted every 6 months.

### Ethics statement

The study protocol was approved by the Institutional Review Board of Kaohsiung Medical University Hospital (KMUHIRB-E(II)-20150089). Written informed consent was obtained from all patients, and all clinical investigations were conducted according to the principles expressed in the Declaration of Helsinki. The patients provided consent for the publication of clinical details.

### Outcomes

The association of early and late mTORi initiation with major transplant outcomes in kidney transplant recipients was analyzed, which included overall survival, patient survival, first-year rejection, and PTMs.

### Statistical analysis

The baseline characteristics of all the patients are expressed as percentages for categorical data and means ± standard deviations for normally distributed continuous variables. Multivariate logistic regression analyses were performed to evaluate the association among mTORi, rejection, and PTM. Multivariate Cox regression analysis was conducted to evaluate the association between mTORi and graft failure and mortality. *P* < 0.05 was considered statistically significant. All analyses were performed using STATA statistical software, v11 (StataCorp LP, College Station, TX, USA). Graft and patient survival estimates were compared using the log-rank test. Kaplan-Meier estimates of graft/patient survivals were compared using the log-rank test. Hazard ratios for graft failure/mortality were calculated using the multivariate Cox regression analysis. Odds ratios for rejection/malignancy were examined using multivariate logistic regression analysis. Adjustments were made for the following major recipient factors: age, body mass index, diabetes mellitus, cause of end-stage renal disease, hepatitis B virus (HBV), hepatitis C virus (HCV), and immunosuppression.

## Results

The baseline characteristics and demographic data of the study cohort are presented in Table 1. A total of 201 kidney transplant recipients were included, with a mean age of 46.2 ± 11.6 years and body mass index of 22.3 ± 3.7 kg/m^2^. Of the included patients, 51.7% were men, and 11.6% and 7% were HBV and HCV carriers, respectively. The mean follow-up duration was 2368 ± 1700 days. The mean duration of mTORi use was 821.3 ± 3225 days (Table 1).

The mean creatinine level at conversion was 1.46 ± 0.48 mg/dL and 1.30 ± 0.53 mg/dL for the early and late mTORi groups, respectively (Table 1). Most patients received the interleukin-2 receptor antagonist Simulect^®^ (Novartis Pharma Stein AG, Switzerland) as the induction agent (84.9%). The maintenance regimens consisted mostly of tacrolimus (84.5%) and mycophenolate mofetil (MMF, 93.0%). Of the included patients, 16.9% had steroid-free maintenance treatment for 3 months after transplantation (Table 1). The overall mortality rate was 11.4%, mainly due to cardiovascular events, followed by PTM and infection (Table 2).

Of the 201 patients, 28 (13.9%) developed PTM, 15 of whom (19.2%) were mTORi nonusers. The most common PTM was urothelial carcinoma (n = 10), followed by hepatocellular carcinoma (n = 7) (Table 3).

**Figure 1:**
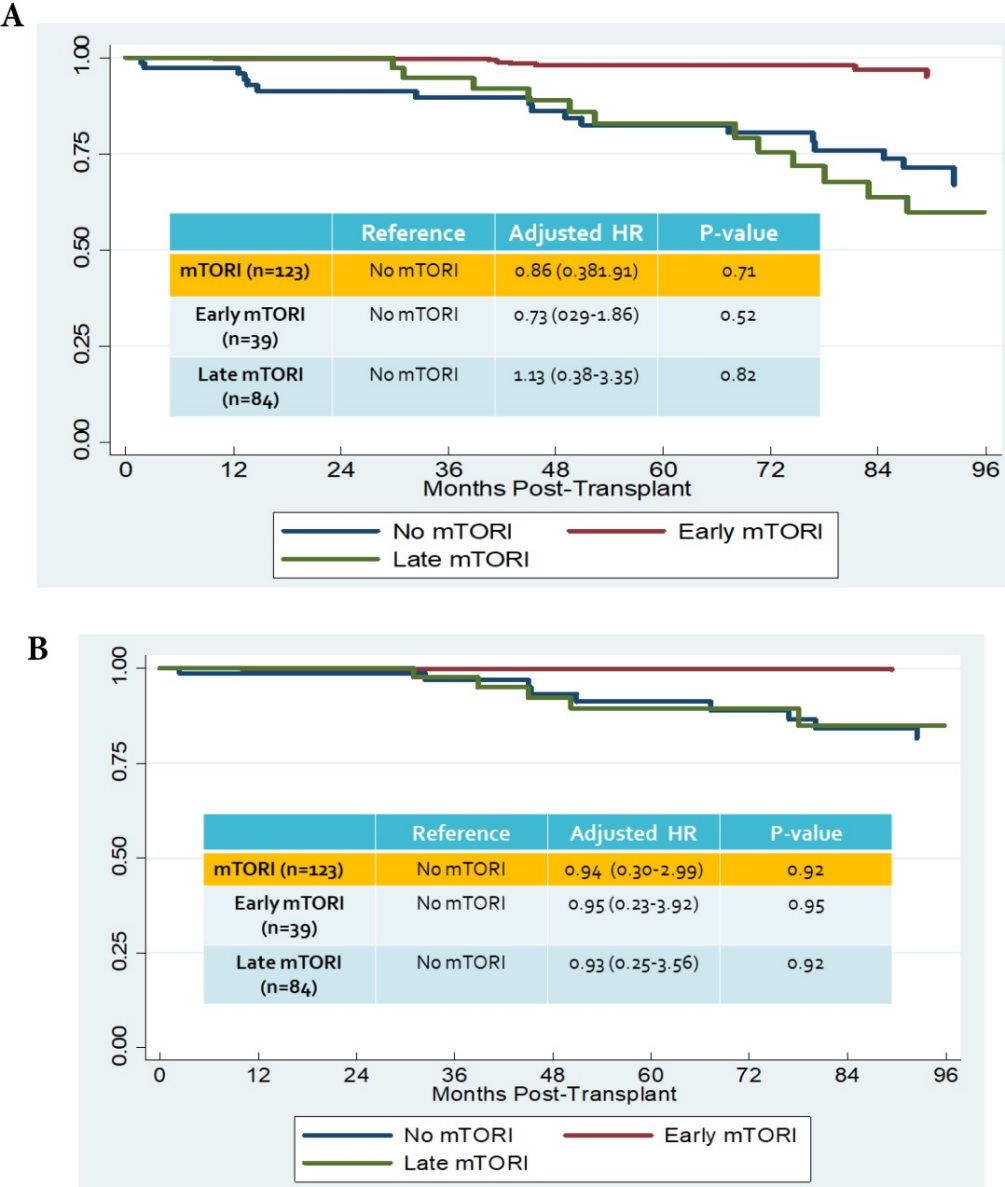
A. Overall graft survival in mTORI User and Non-users. Figure 1B. Patients survival in mTORI User and Non-users.

**Figure 2:**
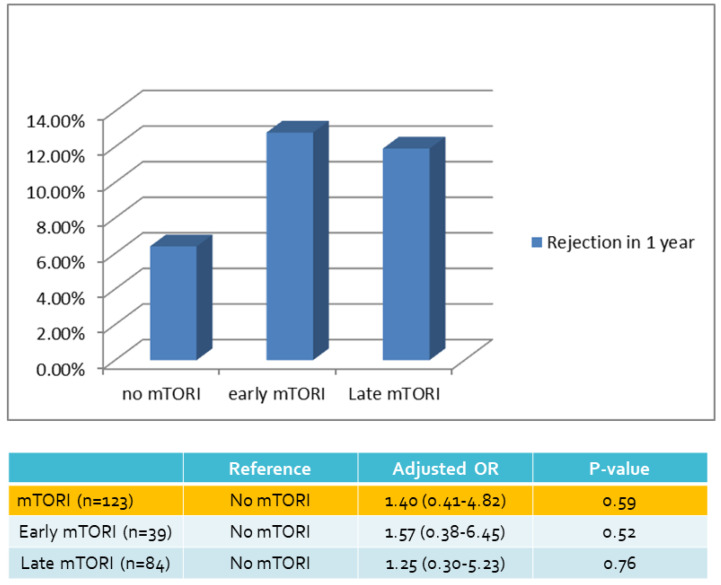
Rejection Rate in 1 year of mTORI User and Non-users.

**Figure 3:**
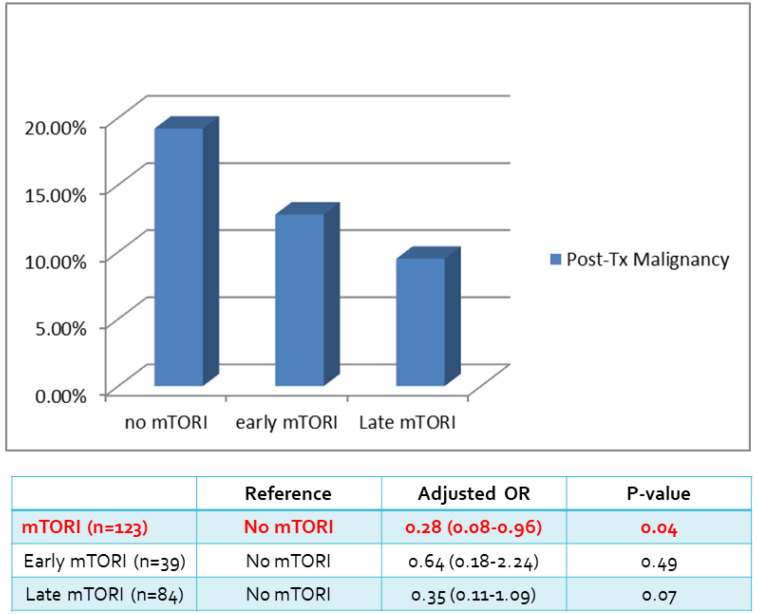
Post-transplant Malignancy rate in in mTORI User and Non-users.

We compared overall graft and patient survival with and without mTORi use. In the multivariate analysis, no significant association was noted between these two groups, but the early mTORi group exhibited a trend of better survival outcomes than did the other groups (Figure 1A, 1B). mTORi users had higher incidents of graft rejection 1 year after transplantation, but this association was not significant in multivariate analysis (adjusted OR: 1.40, *P* = 0.59; Figure 2). After adjustment for confounding factors, mTORi users had a lower incidence of PTM than did nonusers (adjusted OR: 0.28, *P* = 0.04), but no significant difference was observed between early and late mTORi users (Figure 3).

## Discussion

In this study, we examined the timing of mTORi usage and its related outcomes and reviewed the incidence of PTM in kidney transplant recipients. mTORi users had a lower incidence of PTM than did nonusers. However, no differences were observed between early and late mTORi users in patient survival, graft survival, or acute rejection.

mTORi exert complex effects on the immune system due to the multiple roles of the mTOR signaling pathway in the immune cascade 20. mTOR pathway inhibition interferes with cell growth and proliferation and suppresses the immune response by interfering with T-cell proliferation 21, 22. Compared with CNIs, mTORi have less direct nephrotoxicity. mTORi combined with low-dose cyclosporine lowers skin cancer incidence 23, 24. In the CONVERT trial, kidney transplant recipients with sirolimus and a CNI-free regimen had a lower overall incidence of malignancies (2.1 vs 6.0 malignancies per 100 person-years of exposure) 23. However, in the ZEUS study, which compared everolimus with CNI elimination versus a standard CNI regimen, malignancy was reported in 1.6% of everolimus-treated patients compared with 6.4% in the standard CNI group 25. These results imply that mTORi provide protection against PTM.

Compared with the general population, kidney transplant recipients have a higher risk of cancer, especially skin cancer and virus-related cancers 1, 29. Cancers with higher standardized incidence ratios observed in organ transplant recipients are nonmelanoma skin cancers, followed by Kaposi sarcoma and posttransplant lymphoproliferative disease 26. Urothelial carcinoma is the most common PTM in Taiwan 27, 28. The most common PTMs in our study were urothelial carcinoma and hepatocellular carcinoma. In a nationwide cohort population-based study, Tsai et al. demonstrated that heart, lung, kidney, and liver transplant recipients had a three-fold risk of de novo cancer compared with the general population 29. Kidney transplant recipients were associated with a standardized incidence ratio of 10.93 (95% CI, 9.20-12.99) for urinary tract malignancies, among which bladder cancer was the most common 29. The high prevalence of HBV and HCV infection in Taiwan may explain the increased risk of hepatocellular carcinoma in organ transplant recipients, but this association requires further exploration by comparing kidney transplant recipients with and without HBV and HCV.

The mTORi-based immunosuppressive regimens were introduced in CNI minimization/withdrawal strategies to prevent CNI-associated nephrotoxicity. Early conversion of CNI to mTORi within the first 6 months of transplantation has been proposed as a potential strategy to reduce CNI toxicity 30. However, an increased risk of graft loss was observed in mTORi-treated patients. In the ZEUS study, patients were randomized to everolimus or cyclosporine arms at 4.5 months post-transplant, and a higher biopsy-confirmed acute rejection (BCAR) was observed in the everolimus arm 31,32. In the CONCEPT study in which patients were randomized at 3 months, the BCAR rate was 17% in the sirolimus arm compared with 8% in the cyclosporine arm. Most rejection occurred in the sirolimus arm when steroids were discontinued at 8 months after transplantation 33. To investigate the effect of late conversion of mTORi (>6 months post-transplantation), the CONVERT trial revealed no significant difference in renal benefit, BCAR, graft, and patient survival 34. In our study, minimization in the CNI dosage was noted in the mTORi user group, but this was not significantly associated with patients' survival, graft survival, or acute rejection.

This study has several limitations. First, it was a single-center observational study with a small cohort. Second, the follow-up period was short, which might not reflect longer-term graft outcomes and PTM incidence. Third, sirolimus and everolimus were studied as a class (mTORi) due to the small number of everolimus used. Finally, the modification of immunosuppressive agents in kidney transplant recipients might affect this study's ability to correlate the effect of the dose effect of immunosuppressive agents on PTM incidence.

## Conclusion

In our cohort of renal transplant recipients, mTORi use was associated with a decreased risk of PTM. However, no difference in mortality, graft loss, and PTM was noted in patients with early conversion to mTORi (within 6 months of kidney transplantation). The most common PTMs were urothelial and hepatocellular carcinoma. The discrepancy in cancer occurrence highlighted the need for developing specific strategies for cancer surveillance in kidney transplant recipients.

## Figures and Tables

**Table 1:** Baseline Characteristic and Demographic Data of the mTORI User and Non-users

	No mTORI (N=78)	Early mTORI (N=39)	Late mTORI (N=84)	Total (N=201)
Age at Tx, years	46.8±10.7	45.8±12.4	45.8±12.1	46.2±11.6
Male, %	46.2%	48.7%	58.3%	51.7%
BMI, Kg/m^2^	21.6±2.8	22.3±4.3	22.9±4.1	22.3±3.7
Initial Cr, mg/dl	-	1.46±0.48	1.30±0.53	-
HBV	9 (12.7%)	3 (7.9%)	10 (12.5%)	22 (11.6%)
HCV	7 (10.1%)	3 (7.9%)	3 (3.8%)	13 (7%)
DM	12 (15.4%)	12 (30.7%)	18 (21.4%)	42 (20.9%)
Hypertension	63 (80.8%)	30 (76.9%)	60 (71.4%)	153 (76.1%)
Living donor	7 (9.0%)	4 (12.3%)	3 (3.6%)	14 (7.0%)
Induction	51 (78.5%)	31 (91.2%)	59 (88.1%)	141 (84.9%)
Tacrolimus	69 (89.6%)	36 (92.3%)	64 (76.2%)	169 (84.5%)
MMF	67 (85.9%)	39 (100%)	81 (96.4%)	187 (93.0%)
Steroid-free in 3 m	6 (8%)	14 (35.9%)	14 (17.1%)	34 (16.9%)
Follow-up days	2420±1993	1643±1072	2656±1555	2368±1700
Overall graft failure	27 (34.6%)	7(18%)	18(21.4%)	52 (25.9%)
Back to dialysis	17 (21.8%)	4 (10.3%)	11 (13.1%)	32 (15.9%)
Mortality	11 (14.1%)	3 (7.7%)	9 (10.7%)	23 (11.4%)
Rejection in 1 yr	5 (6.4%)	5 (12.8%)	10 (11.9%)	20 (10.0%)
Post-Tx Malignancy	15 (19.2%)	5 (12.8%)	8 (9.5%)	28 (13.9%)

Abbreviations: Tx: transplant; BMI, body mass index; Cr, creatinine; HBV, hepatitis B; HCV, hepatitis C; DM, diabetes mellitus; MMF, mycophenolate mofetil; m, month; yr, year.

**Table 2:** Causes of Death between mTORI users and Non-users

	No mTORI (N=78)	Early mTORI (N=39)	Late mTORI (N=84)	Total (N=201)
Cardiovascular	4	2	3	9
Infection	2	0	3	5
Malignancy	3	1	3	7
Others	2	0	0	2
All	11	3	9	23

**Table 3:** Types of Malignancy

Type of malignancy	Frequency
Breast cancer	1
Colon cancer	1
Cervix cancer	1
Hepatocellular carcinoma	7
Lung cancer	2
Lymphoma	1
Pancreas cancer	1
Prostate cancer	2
Renal cell carcinoma	1
Urothelial carcinoma	10
Peritoneal carcinomatosis	1
Total	28

## References

[1] Engels EA, Pfeiffer RM, Fraumeni JF Jr, Kasiske BL, Israni AK, Snyder JJ (2011). Spectrum of cancer risk among US solid organ transplant recipients. JAMA.

[2] Grulich AE, van Leeuwen MT, Falster MO, Vajdic CM (2007). Incidence of cancers in people with HIV/AIDS compared with immunosuppressed transplant recipients: a meta-analysis. Lancet.

[3] Kiberd BA, Rose C, Gill JS (2009). Cancer mortality in kidney transplantation. Am J Transplant.

[4] Agraharkar ML, Cinclair RD, Kuo YF, Daller JA, Shahinian VB (2004). Risk of malignancy with long-term immunosuppression in renal transplant recipients. Kidney Int.

[5] Hoshida Y, Tsukuma H, Yasunaga Y, Xu N, Fujita MQ, Satoh T (1997). Cancer risk after renal transplantation in Japan. Int J Cancer.

[6] Hwang JK, Moon IS, Kim JI (2011). Malignancies after kidney transplantation: a 40-year single-center experience in Korea. Transpl Int.

[7] Cheung CY, Lam MF, Chu KH, Chow KM, Tsang KY, Yuen SK (2012). Malignancies after kidney transplantation: Hong Kong renal registry. Am J Transplant.

[8] Xiao J, Zhu X, Hao GY, Zhu YC, Hou HJ, Zhang J (2011). Association between urothelial carcinoma after kidney transplantation and aristolochic acid exposure: the potential role of aristolochic acid in HRas and TP53 gene mutations. Transplant Proc.

[9] Wang LJ, Wong YC, Huang CC (2010). Urothelial carcinoma of the native ureter in a kidney transplant recipient. J Urol.

[10] Maluccio M, Sharma V, Lagman M, Vyas S, Yang H, Li B (2003). tacrolimus enhances transforming growth factor-beta1 expression and promotes tumor progression. Transplantation.

[11] Herman M, Weinstein T, Korzets A, Chagnac A, Ori Y, Zevin D (2001). Effect of cyclosporin A on DNA repair and cancer incidence in kidney transplant recipients. J Lab Clin Med.

[12] Lim WH, Eris J, Kanellis J, Pussell B, Wiid Z, Witcombe D (2014). A systematic review of conversion from calcineurin inhibitor to mammalian target of rapamycin inhibitors for maintenance immunosuppression in kidney transplant recipients. Am J Transplant.

[13] Campbell SB, Walker R, Tai SS, Jiang Q, Russ GR (2012). Randomized controlled trial of sirolimus for renal transplant recipients at high risk for nonmelanoma skin cancer. Am J Transplant.

[14] Xie X, Jiang Y, Lai X, Xiang S, Shou Z, Chen J (2015). mTOR inhibitor versus mycophenolic acid as the primary immunosuppression regime combined with calcineurin inhibitor for kidney transplant recipients: a meta-analysis. BMC Nephrol.

[15] Ekberg H, Bernasconi C, Tedesco-Silva H, Vitko S, Hugo C, Demirbas A (2009). Calcineurin inhibitor minimization in the Symphony study: observational results 3 years after transplantation. Am J Transplant.

[16] Srinivas TR, Schold JD, Guerra G, Eagan A, Bucci CM, Meier-Kriesche HU (2007). Mycophenolate mofetil/sirolimus compared to other common immunosuppressive regimens in kidney transplantation. Am J Transplant.

[17] Isakova T, Xie H, Messinger S, Cortazar F, Scialla JJ, Guerra G (2013). Inhibitors of mTOR and risks of allograft failure and mortality in kidney transplantation. Am J Transplant.

[18] Wolf S, Hoffmann VS, Habicht A, Kauke T, Bucher J, Schoenberg M (2018). Effects of mTOR-Is on malignancy and survival following renal transplantation: A systemic review and meta-analysis of randomized trials with a minimum follow-up of 24 months. PLoS One.

[19] Hahn D, Hodson EM, Hamiwka LA, Lee VW, Chapman JR, Craig JC (2019). Target of rapamycin inhibitors (TOR-I; sirolimus and everolimus) for primary immunosuppression in kidney transplant recipients. Cochrane Database Syst Rev.

[20] Cobbold SP (2013). The mTOR pathway and integrating immune regulation. Immunology.

[21] Lee RA, Gabardi S (2012). Current trends in immunosuppressive therapies for renal transplant recipients. Am J Health Syst Pharm.

[22] Halloran PF (2004). Immunosuppressive drugs for kidney transplantation. N Engl J Med.

[23] Alberu J, Pascoe MD, Campistol JM, Schena FP, Rial Mdel C, Polinsky M (2011). Lower malignancy rates in renal allograft recipients converted to sirolimus-based, calcineurin inhibitor-free immunotherapy: 24-month results from the CONVERT trial. Transplantation.

[24] Campistol JM, Eris J, Oberbauer R, Friend P, Hutchison B, Morales JM (2006). Sirolimus therapy after early cyclosporine withdrawal reduces the risk for cancer in adult renal transplantation. J Am Soc Nephrol.

[25] Luan FL, Hojo M, Maluccio M, Yamaji K, Suthanthiran M (2002). Rapamycin blocks tumor progression: unlinking immunosuppression from antitumor efficacy. Transplantation.

[26] Vajdic CM, van Leeuwen MT (2009). Cancer incidence and risk factors after solid organ transplantation. Int J Cancer.

[27] Li WH, Chen YJ, Tseng WC, Lin MW, Chen TJ, Chu SY (2012). Malignancies after renal transplantation in Taiwan: a nationwide population-based study. Nephrol Dial Transplant.

[28] Lee KF, Tsai YT, Lin CY, Hsieh CB, Wu ST, Ke HY (2016). Cancer Incidence among Heart, Kidney, and Liver Transplant Recipients in Taiwan. PLoS One.

[29] Tsai HI, Lee CW, Kuo CF, See LC, Liu FC, Chiou MJ (2017). De novo malignancy in organ transplant recipients in Taiwan: a nationwide cohort population study. Oncotarget.

[30] de Fijter JW, Holdaas H, Øen O, Sanders J-S, Sundar S, Bemelman FJ (2017). Early Conversion From Calcineurin Inhibitor- to Everolimus-Based Therapy Following Kidney Transplantation: Results of the Randomized ELEVATE Trial. Am J Transplant.

[31] Budde K, Lehner F, Sommerer C, Arns W, Reinke P, Eisenberger U (2012). Conversion from cyclosporine to everolimus at 4.5 months posttransplant: 3-year results from the randomized ZEUS study. Am J Transplant.

[32] Sommerer C, Budde K, Zeier M, Wuthrich RP, Reinke P, Eisenberger U (2016). Early conversion from cyclosporine to everolimus following living-donor kidney transplantation: outcomes at 5 years posttransplant in the randomized ZEUS trial. Clin Nephrol.

[33] Lebranchu Y, Thierry A, Toupance O, Westeel PF, Etienne I, Thervet E (2009). Efficacy on renal function of early conversion from cyclosporine to sirolimus 3 months after renal transplantation: concept study. Am J Transplant.

[34] Schena FP, Pascoe MD, Alberu J, del Carmen Rial M, Oberbauer R, Brennan DC (2009). Conversion from calcineurin inhibitors to sirolimus maintenance therapy in renal allograft recipients: 24-month efficacy and safety results from the CONVERT trial. Transplantation.

